# Safety Assessment of Microbicide 2P23 on the Rectal and Vaginal Microbiota and Its Antiviral Activity on HIV Infection

**DOI:** 10.3389/fimmu.2021.702172

**Published:** 2021-08-10

**Authors:** Zhengqin Gao, Rui Fu, Xiaobo Li, Ji Wang, Yuxian He

**Affiliations:** ^1^NHC Key Laboratory of Systems Biology of Pathogens, Institute of Pathogen Biology and Center for AIDS Research, Chinese Academy of Medical Sciences and Peking Union Medical College, Beijing, China; ^2^Institute for Laboratory Animal Resources, National Institutes for Food and Drug Control, Beijing, China

**Keywords:** HIV, membrane fusion inhibitor, inflammatory cytokines, microbiota, mucosal immune system

## Abstract

Containment of the AIDS pandemic requires reducing HIV transmission. HIV infection is initiated by the fusion of the membrane between the virus and the cell membrane of the host. 2P23 is an effective HIV membrane fusion inhibitor that may be a good entry inhibitor microbicide candidate. This study evaluated the potential of using gel-formulated 2P23 as a topical microbicide to prevent sexual transmission of HIV in the rectum and vagina. Our data revealed that 2P23 formulated in gel is effective against HIV. There was no change in antiviral activity at 25°C for 4 months or 60°C for 1 week. In addition, we demonstrated that the 2P23 gel was stable and fully functional at pH 4.0–8.0 and under different concentrations of H_2_O_2_. Finally, the 2P23 gel exhibited no cytotoxicity or antimicrobial activity and did not induce inflammatory changes in the rectal or vaginal mucosal epithelium in New Zealand rabbits after 20 mg/day daily rectovaginal application for 14 consecutive days. Despite repeated tissue sampling and 2P23 gel treatment, the inflammatory cytokines and microbiota of the rectum and vagina remained stable. These results add to general knowledge on the *in vivo* evaluation of anti-HIV microbicide application concerning inflammatory cytokines and microbiota changes in the rectum and vagina. These findings suggest that the 2P23 gel is an excellent candidate for further development as a safe and effective pre-exposure prophylactic microbicide for the prevention of HIV transmission.

## Introduction

According to the latest global statistics report, 38 million individuals are living with HIV (1). Although 26 million patients received antiretroviral (ARV) treatment as of the end of June 2020, treatment efficacy is largely limited by daily medication, poor adherence, and the lifelong burden and stigma associated with side effects, such as kidney failure and bone loss (2, 3). It is well known that HIV transmission is primarily mediated *via* sexual contact. The rectal epithelium is composed of single-layered columnar cells and is a desired target for HIV viral penetration (4, 5). Preventing HIV transmission is of great importance, especially in individuals with a high risk of HIV exposure.

Modifying sexual behaviors and the use of condoms are fundamental prevention strategies for HIV transmission. Condoms are effective in preventing the transmission of HIV (6–8). However, consistent condom use is not optimal, and controls are lax. In addition, among the Ontario cohort of men who had sex with men (MSM), 693 were infected with HIV, and 51% (95% confidence interval, 17%–77%) used condoms. These data indicate that despite the efficacy of condoms, condom failure occurs in gay men exposed to HIV (9).

In addition to condoms, microbicides are also applied for HIV prevention. To facilitate application to the rectum and vagina, microbicides are usually used in the form of lubricants, creams, gels, films, capsules, sponges, rings, tablets, electrospun fibers, suppositories, lavage, or enemas to topically prevent HIV from entering and/or replicating in mucosal cells (10–12). Topical pre-exposure prophylaxis (PrEP) is a microbicide that inhibits viral infection at the mucosal level (13, 14). The antiviral activity of PrEP is dependent on the antiretroviral regimen. Therefore, accumulation of antiretroviral drugs might induce side effects and have to be stopped. As a consequence, the antiviral property of PrEP is abrogated (15–22).

To maintain a sustained preventive effect, we synthesized a 2P23 peptide from the C-terminal heptapeptide repeat region (CHR) of the HIV fusion protein gp41 (23). In this study, we further assessed the activity and safety of 2P23 for blocking HIV infection. Herein, we report that 2P23 is a novel membrane fusion inhibitory peptide with exceptional potency against HIV infection in gel formulation. We also performed safety evaluations, assessing its cytotoxicity and antibacterial potential *in vitro*, and analyzed the inflammatory response and microbiota changes *in vivo*. Collectively, our data demonstrate the potential of 2P23 membrane fusion inhibitors as new antiviral agents for the prevention of HIV infection.

## Materials and Methods

### Ethics Statement

All animal experiments were conducted in accordance with the guidelines and protocols approved by the Ethics Committee for Animal Experimentation of the National Institute for Food and Drug Control (NIFDC; Beijing, China) [protocol No. 2019 (B) 010]. To ensure personnel safety and animal welfare, research on animals was performed in strict accordance with the highest scientific, humane, and ethical principles concerning experiments set out in the Guidelines for the Care and Use of Laboratory Animals.

### Peptide Synthesis

Peptides were synthesized on rink amide 4-methylbenzhydrylamine (MBHA) resin using standard solid phase 9-fluororenyl methoxycarbonyl (FMOC) chemistry at SciLight Peptide Biological Technology Co., Ltd. (SciLight Peptide; Beijing, China) and analyzed by high-performance liquid chromatography (HPLC) and matrix-assisted laser desorption ionization time of flight mass spectrometry (MALDI-TOF MS) to determine that the purity of the peptide was >95%. The concentration of the peptide was measured by UV absorbance and theoretically calculated by the molar extinction coefficients of tryptophan and tyrosine residues.

### Therapeutically Active Compound

2P23 is a novel short peptide (23 mer) fusion inhibitor with an M-T hook structure, HIV-2 sequence and salt bridge formation residue, and a molecular weight of 3717.96 g/mol (2P23 amino acid sequence: EMTWEEWEKKVEELEKKIEELLK). 2P23 is a highly stable helical peptide that binds highly to surrogate targets from HIV-1, HIV-2, and simian immunodeficiency virus (SIV) and is effective at inhibiting HIV-1 and HIV-2 replication (mean IC_50_ for HIV-1, 5.57 nM; mean IC_50_ for HIV-2, 15.38 nM) (23).

### Virus Source and Cell Culture

The Global HIV-1 ENV Cloning Group was obtained from David Montefiori through the NIH AIDS Reagent Program (AIDS Research and Reference Reagent Program, NIH, USA), Division of AIDS, NIAID, NIH. TZM-bl indicator cells were obtained from Dr. John C. Kappes, Dr. Xiaoyun Wu, and Tranzyme Inc (24–29), which stably express large amounts of CD4 and CCR5 with endogenous expression of CXCR4 and were maintained in complete growth medium composed of DMEM (HyClone™, GE Healthcare, Utah, USA) supplemented with 10% heat-inactivated FBS (Gibco, Grand Island, NY, USA), 100 IU/ml penicillin, and 100 μg/ml streptomycin (HyClone). HEK293T cells and the human T-cell line MT-4 were purchased from the American Type Culture Collection (ATCC; Manassas, USA). Peripheral blood mononuclear cells (PBMCs) from healthy individuals were separated by Ficoll-Paque Plus (GE Healthcare, Waukesha, USA) with density gradient centrifugation and stimulated with 5 μg/ml PHA (Sigma-Aldrich, St. Louis, USA) for 72 h.

### Inhibitory Activity of Membrane Fusion Inhibitory Peptide on HIV

The HIV membrane fusion inhibition peptide sensitivity test was performed by polyethylenimine (PEI) transfection to produce env-specific pseudoviruses. Briefly, 293T cells were cotransfected with env-deficient pSG3Δenv plasmid DNA and pcDNA3.1-env plasmid containing the full-length env gene derived from plasma virus. The supernatant was collected, aliquoted, and stored at −80°C until use. The titers of these viruses were determined by infecting TZM-bl cells, and the relative luminescence unit (RLU) readings were recorded using a Veritas microplate luminometer. The cutoff value was threefold higher than that of the cell control well, and 50% tissue culture infectious doses (TCID_50_) of the virus were calculated using the Reed and Muench method (30). To determine the peptide inhibitor dose corresponding to the 50% inhibitory concentration (IC50) with a confidence interval of 95%, a piecewise linear dose–response curve was constructed for each virus type. After establishing a dose–response curve, the IC50 dose was estimated using appropriate correction techniques using GraphPad Prism statistical software. Under the linear assumption, the standard error of IC50 can be estimated (31).

### Hydroxyethyl Cellulose

Hydroxyethyl cellulose (HEC) (catalog# 434973) was purchased from Sigma-Aldrich (St. Louis, MO, USA). The HEC gel is a “universal” gel that has shown sufficient stability in gel formulation, as previously described (32–34), and is safe and sufficiently inactive to be used in clinical studies of experimental microbicides. In our study, the 2P23 gel dosage formulation was prepared using the gel method, and HEC was selected as the gelling agent. Aqueous systems containing decreasing concentrations (%) of HEC were prepared to evaluate HEC cytotoxicity in TZM-bl cells using the Cell Counting Kit-8 (CCK-8; Kumamoto Michio, Japan) according to the manufacturer’s instructions.

Cytotoxicity was evaluated by calculating the cell viability = (OD_450_ of cell-HEC group − OD_450_ of blank control group)/(OD_450_ of HEC-free cell control group − OD_450_ of blank control group) × 100%. The evaluation criteria were derived from the *Biological evaluation of medical devices*—*Part 5: Test for in vitro cytotoxicity* (GB/T 16886.5-2017/ISO 10993-5:2009, IDT) (35).

### Products

The 2P23 peptide in powder form was provided by Scilight Peptide (Beijing, China). A solution of 2P23 peptide was prepared in PBS. 2P23 gels consist of 2P23 peptide solutions of different concentrations added to a formulation containing a gel (HEC), glycerin, and the preservative methyl 4-hydroxybenzoate, filtered through a 0.45-μm microporous membrane. Formulation of the vehicle control gel was the same but without the active ingredient (2P23).

### Physicochemical Property Testing

The primary physicochemical parameters commonly used to evaluate semisolids include viscosity, osmotic pressure, and pH. The viscosity was determined using the CP41 spindle on a Wells/Brookfield™ cone plate Brookfield Model DVIII viscometer (Brookfield Eng. Lab., Inc., MA, USA). Data were collected using Rheocalc software (Brookfield Eng. Lab., Inc., MA, USA). To compare data from different samples, viscosity values obtained at 50 s−1 were used in the analysis. The shear stress (dyne/cm2) was best fitted by the Bingham equation using Rheocalc software. pH was determined using a Mettler-Toledo SevenExcellence™ Multiparameter pH meter (Mettler) with a Mettler-Toledo InLab^®^ Expert Pro-ISM (Mettler) probe calibrated using three points, pH 4.01, 6.86, and 9.18. Osmolality was determined using a SMC30D osmometer (TianJin TianHe Analytic Instrument Co., Ltd, Tianjin, China) calibrated using GBW(E) 198.5, 600.8, and 1000 mOsmol/kg calibration standards (National Institute of Metrology, Beijing, China).

### Cytotoxicity Test of the 2P23 Gel *In Vitro*

The toxicity of the 2P23 gel against PBMCs, CEMss-CCR5, MT-4, and TZM-bl cells was determined using a CCK-8 assay. Cytotoxicity was evaluated by calculating the cell viability = (OD_450_ of cell-2P23 gel group − OD_450_ of blank control group)/(OD_450_ of 2P23 gel-free cell control group − OD_450_ of blank control group) × 100%. The evaluation criteria were derived from the *Biological evaluation of medical devices*—*Part 5: Test for in vitro cytotoxicity* (GB/T 16886.5-2017/ISO 10993-5:2009, IDT) (35).

### Antibacterial Activity of the 2P23 Gel

The antimicrobial activity of the 2P23 gel was determined according to the Clinical and Laboratory Standards Institute (CLSI) guidelines (36, 37). Microorganisms used in the antibacterial test were *Escherichia coli* ATCC 25922, *Pseudomonas aeruginosa* ATCC 27853, *Enterococcus faecalis* ATCC 29212, and *Staphylococcus aureus* ATCC 25923. Reference microbial strains were purchased from ATCC, and a panel of probiotics strains, including *Lactobacillus rhamnosus*, *Lactobacillus acidophilus*, *Lactobacillus reuteri*, *Lactobacillus fermentium*, *Lactobacillus casei*, *Lactobacillus paracasei*, *Lactobacillus delbrueckii* subsp. *bulgaricus*, *Bifidobacterium lactis*, *Bifidobacterium breve*, *Bifidobacterium bifidum*, and *Bifidobacterium longum*, were purchased from Shanghai Aurinda Health Food Co., Ltd. Broth microdilution minimum inhibitory concentration (MIC) testing was performed for the 2P23 gel according to CLSI M07 and CLSI M100 guidelines. The viability of the bacteria was determined by measuring the optical density (OD) absorbance at 450 nm, as described above. MICs were measured for bactericidal activity. Walch^®^ Instant Hand Sanitizer positive controls (Kills 99.9% of germs) (Whealth Lohmann CENTRALIN GmbH, Germany) were included for each organism–2P23 gel combination.

### Effect of pH and H_2_O_2_ on the Stability and Antiviral Activity of the 2P23 Gel

The effect of pH on the 2P23 gel was evaluated at pH 4.0, 6.0, and 8.0. Briefly, the antiviral activity of 2P23 gels incubated at pH 4.0, 6.0, or 8.0 in complete growth medium at 37°C for 48 h was determined by a single-round viral infection assay using luciferase reporter gene detection in the abovementioned TZM-bl cells. To test the oxidation of H_2_O_2_, the 2P23 gel was treated with 1.2 μM H_2_O_2_ and 5 μM H_2_O_2_ as previously described (38). The antiviral activity of the 2P23 gel in H_2_O_2_ was determined as described above.

### Accelerated Stability and Antiviral Activity of the 2P23 Gel

The accelerated stability and antiviral activity of the 2P23 gel under different temperature and time conditions were studied. In brief, the 2P23 gel was stored at 4°C, 25°C, 40°C, and 60°C for 1 to 24 weeks at 75% relative humidity (RH). Equal portions of the 2P23 gel were taken weekly, diluted in complete medium, and incubated at 37°C for 48 h. The antiviral activity was determined as described above. In addition, the stability of the 2P23 gel was further analyzed by HPLC.

### HPLC Experiment

2P23 in gel or PBS was analyzed by HPLC on an Ultimate3000 (Thermo) liquid chromatograph. A reversed-phase Ultra Aqueous C18 analysis column (2.1 mm × 100 mm, 5-μm particle size) and guard column (2.1 mm × 12.5 mm, 5 μm particle size) were purchased from Agilent. ChemStation software (Agilent) was used to calculate the peak area (Pa), and the recovery rate of 2P23 was calculated as “X week PA sample/0 week PA sample × 100%”.

### Safety Evaluation in Rabbit Model *In Vivo*

Rabbits are an ideal animal model for assessing rectal and vaginal products, as described elsewhere (39, 40). A total of 48 specific pathogen-free (SPF) New Zealand White (NZW) rabbits were provided by the National Center for Rodent Laboratory Animal Resources (Beijing, China). The rabbits were 8 to 9 weeks old and weighed between 1.790 and 2.424 kg. For the *in vivo* safety study, 48 rabbits (24 male rabbits and 24 female rabbits) were randomized into 12 groups (two male rabbits and two female rabbits per group). Rabbits were randomly assigned to 12 experimental groups: placebo control group rabbits receiving PBS only (*n* = 4), vehicle control group rabbits receiving empty gel (*n* = 4), SFT gel high-dose group rabbits receiving gel formulation containing 20 mg/ml SFT (*n* = 4), SFT gel low-dose group rabbits receiving gel formulation containing 4 mg/ml SFT (*n* = 4), 2P23 gel high-dose group rabbits receiving gel formulation containing 20 mg/ml 2P23 (*n* = 4), 2P23 gel low-dose group rabbits receiving gel formulation containing 4 mg/ml 2P23 (*n* = 4), LP80 gel high-dose group rabbits receiving gel formulation containing 20 mg/ml LP80 (*n* = 4), LP80 gel low-dose group rabbits receiving gel formulation containing 2 mg/ml LP80 (*n* = 4), LP98 gel high-dose group rabbits receiving gel formulation containing 20 mg/ml LP98 (*n* = 4), LP98 gel low-dose group rabbits receiving gel formulation containing 2 mg/ml LP98 (*n* = 4), nonionic surfactants irritation control group rabbits receiving 100 mg/ml Triton X-100 (*n* = 4), and anionic surfactants irritation group rabbits receiving 100 mg/ml SDS (*n* = 4) (**Table 1**). Rabbits were dosed intrarectally and intravaginally with active formulation (2P23, SFT, LP80, and LP98), irritation control (Triton X-100, SDS), vehicle control (gel), or placebo control (PBS) using 4.7 mm (14Fr) silicone-rubber catheters (Yangzhou Huayue Technology Development Co., LTD, Yangzhou, China) connected to a 20# lavage apparatus with a syringe without needle. Active formulation compound gel, irritation control, vehicle control, or placebo control were placed in the rectal tract approximately 9 cm away from the anus and in the vaginal canal against the cervix approximately 5 cm away from the cunnus to their respective groups daily for 14 consecutive days. Rectal lavage (RL) and cervical lavage (CVL) were collected by irrigating the rectal walls and cervix and the lateral vaginal walls with PBS before (baseline) and after 24 h of administration of each dose. On the 14th day, rabbits were euthanized, the rectum and vagina were excised, and the middle was cut open. After macroscopic observation, tissue was removed from the upper, middle, and lower segments of the vagina or rectum for histopathological examination. Grading was performed based on Eckstein et al. (39).

**Table 1:** Animal characteristics in the 2P23 gel trial.

Group	ID	Gender	Initial weight	Final weight	ID	Gender	Initial weight	Final weight
PBS	1	Male	2.193 kg	2.452 kg	25	Female	2.242 kg	2.419 kg
PBS	2	Male	2.127 kg	2.382 kg	26	Female	2.136 kg	2.336 kg
Gel	3	Male	2.176 kg	2.495 kg	27	Female	2.141 kg	2.424 kg
Gel	4	Male	1.922 kg	2.162 kg	28	Female	1.836 kg	2.022 kg
2% SFT gel	5	Male	1.990 kg	2.420 kg	29	Female	2.368 kg	2.569 kg
2% SFT gel	6	Male	1.922 kg	2.262 kg	30	Female	2.053 kg	2.324 kg
0.4% SFT gel	7	Male	2.020 kg	2.330 kg	31	Female	1.956 kg	2.080 kg
0.4% SFT gel	8	Male	1.941 kg	2.145 kg	32	Female	2.096 kg	2.335 kg
2% 2P23 gel	9	Male	2.121 kg	2.449 kg	33	Female	2.032 kg	2.325 kg
2% 2P23 gel	10	Male	2.100 kg	2.358 kg	34	Female	1.840 kg	2.055 kg
0.4% 2P23 gel	11	Male	2.145 kg	2.475 kg	35	Female	1.996 kg	2.362 kg
0.4% 2P23 gel	12	Male	2.116 kg	2.356 kg	36	Female	2.248 kg	2.390 kg
2% LP80 gel	13	Male	2.120 kg	2.367 kg	37	Female	2.268 kg	2.443 kg
2% LP80 gel	14	Male	2.424 kg	2.596 kg	38	Female	2.291 kg	2.338 kg
0.2% LP80 gel	15	Male	2.205 kg	2.522 kg	39	Female	2.283 kg	2.462 kg
0.2% LP80 gel	16	Male	2.067 kg	2.273 kg	40	Female	2.427 kg	2.542 kg
2% LP98 gel	17	Male	2.095 kg	2.400 kg	41	Female	2.248 kg	2.434 kg
2% LP98 gel	18	Male	2.249 kg	2.415 kg	42	Female	2.070 kg	2.240 kg
0.2% LP98 gel	19	Male	2.170 kg	2.384 kg	43	Female	2.363 kg	2.482 kg
0.2% LP98 gel	20	Male	2.090 kg	2.292 kg	44	Female	2.348 kg	2.452 kg
10% Triton X-100	21	Male	2.109 kg	2.420 kg	45	Female	2.299 kg	2.465 kg
10% Triton X-100	22	Male	1.823 kg	2.126 kg	46	Female	2.276 kg	2.405 kg
10% SDS	23	Male	1.790 kg	2.116 kg	47	Female	2.109 kg	2.217 kg
10% SDS	24	Male	1.956 kg	2.226 kg	48	Female	2.246 kg	2.419 kg

### Histopathological Analysis of Mucosal Tissues

The rectal and vaginal tissues were fixed for 24 h and evaluated for histopathological changes in mucosal tissues following *in vivo* treatment with 2P23 gel. In each biological sample, the rectal or vaginal epithelium was assessed for lesions, inflammatory infiltrates, vascular congestion, and/or submucosal edema. The evaluation criteria were derived from the *Biological evaluation of medical devices—Part 10: Tests for irritation and skin sensitization* (GB/T16886.10-2017/ISO10993-10:2010, IDT) (40).

### Evaluation of Inflammatory Cytokines

Soluble markers of inflammation in CVL and RL were quantified by enzyme-linked immunosorbent assay (ELISA) using 14 commercially available rabbit cytokine kits (Cloud-Clone Corp., Wuhan, China). IL-4, IL-5, IL-6, IL-8, IL-10, IL-17, IFN-γ, MCP-1, IL-1a, IL-1Ra, ELAM-1, ICAM-1, VEGFA, and PDGFA were included in these 14 cytokines that we tested. A standard curve was generated for each cytokine. The optical density was read using an Infinite^®^ F50 absorbance microplate reader (Tecan). Cytokine concentration was calculated by quadratic regression analysis based on logarithmic transformation optical density.

### Rectal and Vaginal Microbiota Analysis

#### Sample Collection

RL and CVL samples were collected on September 10, 2019, and daily from September 17 to 30, 2019. To control for potential variants, RL and CVL samples were randomly chosen as follows (1): T0 (baseline): CM-01 (NWR1), CM-02 (NWR2), CF-01 (NWF1), and CF-02 (NWF2); (2) T14 (after 14 days administration): PBS group: PBSM1, PBSM2, PBSF25, and PBSF26; gel group: GELM3, GELM4, GELF27, and GELF28; SFT gel high-dose group: SFTHM5, SFTHM6, SFTHF29, and SFTHF30; SFT gel low-dose group: SFTLM7, SFTLM8, SFTLF31, and SFTLF32; 2P23 gel high-dose group: 2P23HM9, 2P23HM10, 2P23HF33, and 2P23HF34; 2P23 gel low-dose group: 2P23LM11, 2P23LM12, 2P23LF35, and 2P23LF36; LP80 gel high-dose group: LP80HM13, LP80HM14, LP80HF37, and LP80HF38; LP80 gel low-dose group: LP80LM15, LP80LM16, LP80LF39, and LP80LF40; LP98 gel high-dose group: LP98HM17, LP98HM18, LP98HF41, and LP98HF42; LP98 gel low-dose group: LP98LM19, LP98LM20, LP98LF43, and LP98LF44; Triton X-100 group: Triton100M21, Triton100M22, Triton100F45, and Triton100F46; SDS group: SDSM23, SDSM24, SDSF47, and SDSF48.

### DNA Extraction

The total community genomic DNA of the RL and CVL samples was extracted using the E.Z. N. A Soil DNA Kit (Omega, USA) according to the manufacturer’s instructions. The concentration of the DNA was determined using Qubit 2.0 (Life, USA).

### 16S rRNA Gene Amplification by PCR

We targeted the V3–V4 hypervariable region of the bacterial 16S rRNA gene. PCR was initiated immediately after DNA extraction. V3–V4 amplicons of the 16S rRNA gene were amplified using Kapa Hifi Hot Start Ready Mix (2 ×) (Takara Bio Inc., Japan). Two common bacterial 16S rRNA gene amplification primers (purified by PAGE) were used: PCR forward primer 341F (5’-CCTACGGGNGGCWGCAC-3’) and PCR reverse primer 805R (5’-GACTACHVGGGTATCTAATCC-3’) (41).

### 16S Gene Library Construction, Quantification, and Sequencing

The free primers and primer dimer species in the amplified products were purified using AmPure XP beads. Samples were sent to Shanghai Sangon Biotechnology Co., Ltd., China, for library construction using Universal Illumina adapters and indexes. Depending on coverage requirements, all the libraries can be pooled for one run. The amplified products in each reaction mixture were aggregated in equal molar ratios according to their concentration. Sequencing was performed using the Illumina MiSeq system (Illumina MiSeq, USA).

### Sequence Processing

After sequencing, the data were collected as follows: (1) PEAR (V0.9.6) software was used to assemble two short Illumina readings based on the overlap and process FASTQ files to generate separate FASTA and QUAL files for analysis using standard methods; (2) sequences with ambiguous bases and lengths greater than 480 base pairs (bp) were removed, and the maximum allowable homopolymer length was 6 bp (42). Sequences shorter than 200 bp were removed; (3) all the same sequences were combined into one; (4) sequences were aligned according to the customized reference database; (5) the integrity of indexes and adapters was checked, and all indexes and adapter sequences were deleted; (6) noise was removed using PRE Cluster tools. Chimera UCHIME was used to detect Chimera. All software was in the mothur package. We resubmitted the valid sequences of each sample to the RDP classifier to identify archaea and bacterial sequences. Species richness and diversity statistics, including coverage, Chao1, Ace, Simpson, and Shannon-Ever, were calculated using mothur. The modified pipeline is described on the mothur website. Finally, all effective bacterial sequences without primers were submitted for data analysis (43).

### Sequencing Data Analysis

Operational taxonomic units (OTUs) were established *de novo* using UCLUST, and 97% sequence homology was truncated (44). OTUs for regions V3–V4 were specified by the Ribosomal Database Project (RDP) Naive Bayes classifier (45, 46). This sequence was compared to the Greengenes core set using the Python nearest alignment space termination (PyNAST) aligner (47). Phylogenetic trees were generated using FastTree, and dilution curves were drawn to calculate alpha and beta diversity of the samples performed by QIIME (48). Similarities between microbial communities were identified using principal coordinate analysis (PCoA), which relies on unweighted and weighted UniFrac. UCHIM software was used to detect and remove chimeric sequences based on the “RDP GOLD” database. OTU cluster analysis was performed according to the Galaxy online platform process. The BIOM file obtained by the QIIME software was uploaded to the Galaxy website for predictive analysis of the Phylogenetic Investigation of Communities by Reconstruction of Unobserved States (PICURSt) functional genes. The information could be obtained by referring to the Kyoto Encyclopedia of Genes and Genomes (KEGG) Orthology class 1 and class 2 functional gene classes to obtain the functional composition of the predicted genome (49).

### Statistical Analysis

All data were derived from at least three separate experiments. All experimental data are expressed as the mean ± standard deviation (Mean ± SD). One-way ANOVA was used, followed by GraphPad Prism (Version 7.00, GraphPad Software, Inc., La Jolla, CA, USA) and Microsoft Excel [Office 365; Microsoft Corp. (MSFT)]. Statistical significance was defined as *p* < 0.05.

## Results

### Cytotoxicity of HEC *In Vitro*

**Figure 1:**
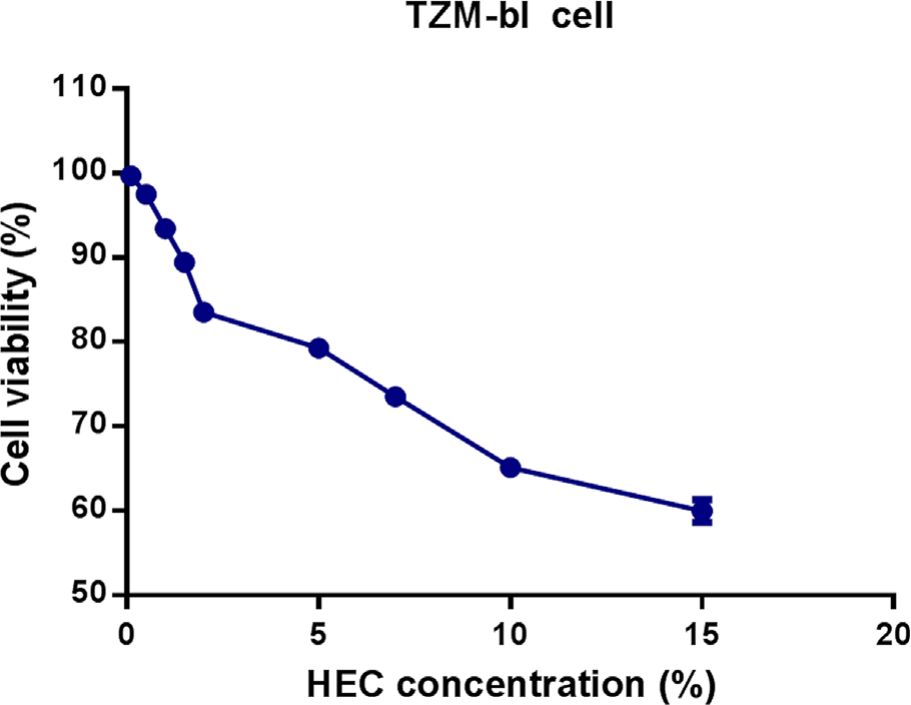
Effects of different concentrations of HEC gel on TZM-bl cell viability. TZM-bl cells were cultured in different concentrations of HEC gel, and cell viability was assessed by CCK-8 assay.

The toxicity of HEC against TZM-bl cells was measured using a CCK-8 assay kit. The survival of TZM-bl cells was assessed under different concentrations of HEC. The results are summarized in **Table 2**. The 0.1% HEC gel, 0.5% HEC gel, 1% HEC gel, 1.5% HEC gel, and 2% HEC gel were level 1, and the cell viability was 99.7%, 97.5%, 93.4%, 89.4%, and 83.5%, respectively. The 5% HEC gel, 7% HEC gel, 10% HEC gel and 15% HEC gel were level 2, and their cell viability values were 79.3%, 73.53%, 65.1% and 60%, respectively. No cytotoxicity (level 0) was defined as cell viability ≥ 100%, minimum cytotoxicity (level 1) was defined as 80% ≤ cell viability <100%, mild cytotoxicity (level 2) was defined as 50% ≤ cell viability < 80%, moderate cytotoxicity (level 3) was defined as 30% ≤ cell viability < 50%, and severe cytotoxicity (level 4) was defined as cell viability < 30%. A grade greater than 2 using this method is considered cytotoxic. A decrease in cell viability greater than 30% is considered cytotoxic. Different concentrations of HEC gel (0.1%, 0.5%, 1%, 1.5%, 2%, 5%, 7%, 10%, and 15% HEC) were screened. According to the evaluation criterion in ISO10993 (35), the cytotoxicity of the 0.1%–2% HEC gel was of ranking 1 and qualified (**Figure 1**). Due to the fluidity and the difficulty of filtration, the 1.5% HEC gel was selected as the final concentration.

**Table 2:** Cytotoxicity of HEC on TZM-bl cells.

Group (*n* = 3)	Cell viability (mean ± SD)	Level*
Cell control	100	0
0.1% HEC-gel	99.66 ± 0.76	1
0.5% HEC-gel	97.49 ± 0.20	1
1.0% HEC-gel	93.43 ± 0.40	1
1.5% HEC-gel	89.43 ± 0.23	1
2.0% HEC-gel	83.54 ± 0.65	1
5.0% HEC-gel	79.28 ± 0.42	2
7.0% HEC-gel	73.52 ± 0.20	2
10.0% HEC-gel	65.13 ± 0.51	2
15.0% HEC-gel	59.98 ± 1.35	2

*Cytotoxicity evaluation: cell viability, no cytotoxicity (level 0) was defined as cell viability ≥ 100%, minimum cytotoxicity (level 1) was defined as 80% ≤ cell viability <100%, mild cytotoxicity (level 2) was defined as 50% ≤ cell viability < 80%, moderate cytotoxicity (level 3) was defined as 30% ≤ cell viability < 50%, and severe cytotoxicity (level 4) was defined as cell viability <30%. A grade greater than 2 by this method is considered cytotoxic. A decrease in cell viability greater than 30% is considered cytotoxic. The evaluation criteria were derived from the Biological evaluation of medical devices—part 5: test for in vitro cytotoxicity (GB/T 16886.5-2017/ISO 10993-5:2009, IDT).

### 2P23 Gel Formulation Evaluation

Formulation tests are required to predict the effect of the product when applied to mucosal surfaces. The primary physicochemical properties commonly assessed for semisolids include osmolality, viscosity, and pH. Osmolality was measured to determine how the drug deviated from isosmolar (290 mOsmol/kg) conditions. This is important because hypertonic products can cause mucosal tissue damage. The osmolality values of the 2P23 gel and its vehicle control gel were 8.8-fold (2,540 mOsmol/kg) and 7.8-fold (2,250 mOsmol/kg), respectively, greater than those under isosmolar conditions. The pH 4.4 or pH 7.4 of both gels was similar to that of the vaginal and rectal environment. The viscosities of the 2P23 gel and its vehicle control gel were 1543 centipoise (cps) and 1316 cps, respectively, at a shear rate of 3 rpm. These results were reproducible in repeated trials.

### The 2P23 Gel Exhibits No Cytotoxicity *In Vitro*

**Figure 2:**
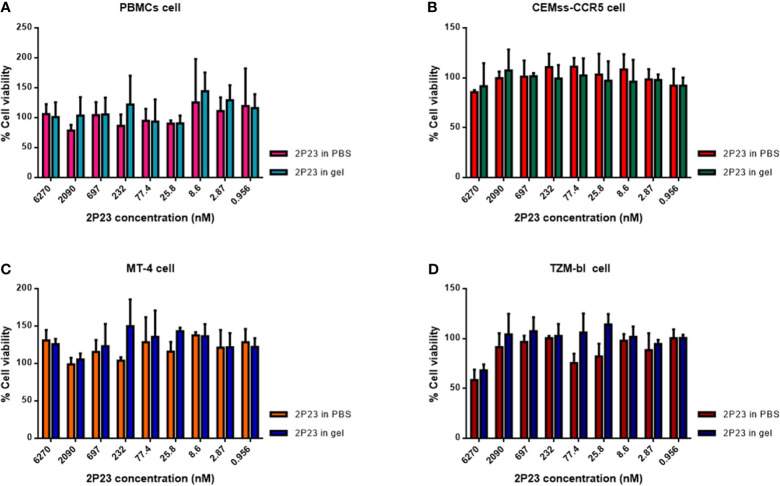
*In vitro* cytotoxicity of the 2P23 gel. The viability of PBMCs **(A)**, CEMss-CCR5 **(B)**, MT-4 **(C)**, and TZM-bl **(D)** cells treated with 2P23 formulated in gel or in PBS at graded concentrations was evaluated by CCK-8 assay.

To determine whether the 2P23 gel exerts cytotoxicity *in vitro*, we incubated PBMCs, CEMss-CCR5, MT-4, and TZM-bl cells with the 2P23 gel at graded concentrations and assessed their cell viability by CCK-8 assay. As illustrated in **Figure 2**, the 2P23 gel exhibited no *in vitro* cytotoxicity to PBMCs, CEMss-CCR5, MT-4, or TZM-bl cells at concentrations as high as 6270 nM, which is approximately 1,126-fold higher than the IC_50_ of 2P23 for inhibiting HIV-1 infection, suggesting that the 2P23 gel has a good safety profile.

### The 2P23 Gel Efficiently Inhibits HIV Infections *In Vitro*

**Figure 3:**
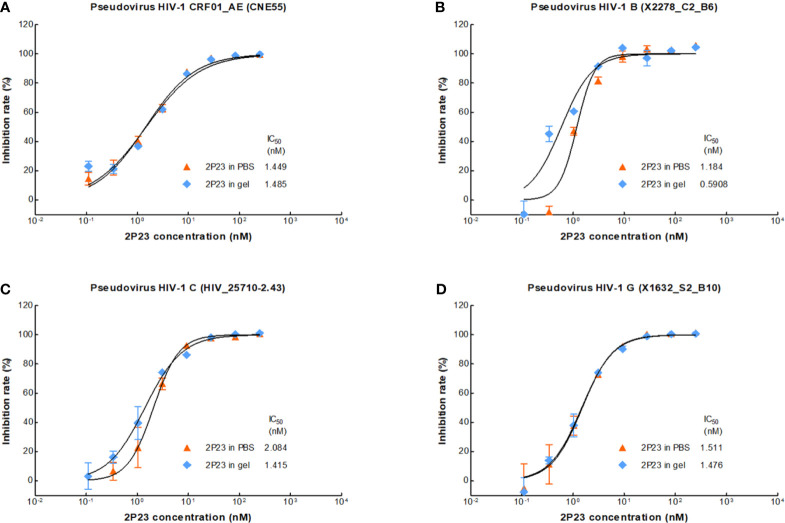
Antiviral activity of the 2P23 gel. 2P23 formulated in gel or in PBS was incubated at 37°C for 48 h The antiviral activity of 2P23 in gel or in PBS was evaluated using a single-round viral infectivity assay using TZM-bl reporter cells and HIV-1 **(A)** pseudotyped HIV-1 CRF01_AE (CNE55), **(B)** pseudotyped HIV-1 B (X2278_C2_B6), **(C)** pseudotyped HIV-1 C (HIV_25710-2.43), and **(D)** pseudotyped HIV-1 G (X1632_S2_B10). The experiments were performed with an initial concentration of 2P23 peptide at 250 nM. These assays were performed in triplicate and repeated three times. Percent inhibition of 2P23 in gel or in PBS and IC_50_ values were calculated as described in the text. Data were expressed as means ± standard deviations (SD).

We therefore formulated 2P23 peptide in gel or in PBS. In our study, 2P23 showed strong broad-spectrum inhibitory activity against HIV-1, such as pseudotyped HIV-1 CRF01_AE (CNE55), B (X2278_C2_B6), C (HIV_25710-2.43), and G (X1632_S2_B10), with the mean IC_50_ values range of 0.5908–2.084 nM (**Figure 3**). To enhance the translational potential of human vaginal and rectal applications, the 2P23 peptide was reformulated in gel rather than PBS to achieve equivalent potency *in vitro*. The 2P23 gel demonstrated full activity against HIV-1, confirming the effective delivery of the 2P23 peptide in the gel formulation.

### The 2P23 Gel Lacks Antibacterial Activity *In Vitro*

*Lactobacillus*, *Bifidobacterium*, *Enterobacter*, and *Enterococcus* are found in a healthy vagina or rectum. They are essential for maintaining an acidic environment (pH 3.5–4.5) or a neutral environment (pH 7.4–8.4) and produce a variety of antiviral and antibacterial substances that inhibit pathogens. Alterations in the normal rectovaginal microbiota can lead to multiple rectovaginal infections and affect the risk of rectovaginal HIV transmission. Therefore, the 2P23 gel must not interfere with normal rectovaginal microbiota. The effects of the 2P23 gel on rectovaginal bacteria were studied using 15 different species of bacteria, namely, *E. coli*, *P. aeruginosa*, *E. faecalis*, *S. aureus*, *L. rhamnosus*, *L. acidophilus*, *L. reuteri*, *L. fermentium*, *L. casei*, *L. paracasei*, *L. delbrueckii* subsp. *bulgaricus*, *B. lactis*, *B. breve*, *B. bifidum*, and *B. longum*, and 2P23 gels did not affect the growth of the bacteria to 2,689,500 nM concentrations (≈ 482,855-fold higher than the IC_50_) (**Table 3**). These studies indicate that 2P23 gels do not affect the normal rectovaginal microbiota.

**Table 3:** *In vitro* anti-bacterial activity of 2P23 gel on *Lactobacillus*, *Bifidobacterium*, *Enterobacter*, and *Enterococcus* species.

Bacteria strain	2P23 gel (MIC*)
*Escherichia coli* ATCC 25922	**>**2,689,500 nM
*Pseudomonas aeruginosa* ATCC 27853	**>**2,689,500 nM
*Enterococcus faecalis* ATCC 29212	**>**2,689,500 nM
*Staphylococcus aureus* ATCC 25923	**>**2,689,500 nM
*Lactobacillus rhamnosus*	**>**2,689,500 nM
*Lactobacillus acidophilus*	**>**2,689,500 nM
*Lactobacillus reuteri*	**>**2,689,500 nM
*Lactobacillus fermentium*	**>**2,689,500 nM
*Lactobacillus casei*	**>**2,689,500 nM
*Lactobacillus paracasei*	**>**2,689,500 nM
*Lactobacillus delbrueckii* subsp. *bulgaricus*	**>**2,689,500 nM
*Bifidobacterium lactis*	**>**2,689,500 nM
*Bifidobacterium breve*	**>**2,689,500 nM
*Bifidobacterium bifidum*	**>**2,689,500 nM
*Bifidobacterium longum*	**>**2,689,500 nM

*MIC, minimal inhibitory concentration.

2P23 gel MIC at 2,689,500 nM, which is ≈482,855-fold higher than the IC_50_.

### Antiviral Activity of the 2P23 Gel Is Preserved at Different pH and H_2_O_2_ Conditions

**Figure 4:**
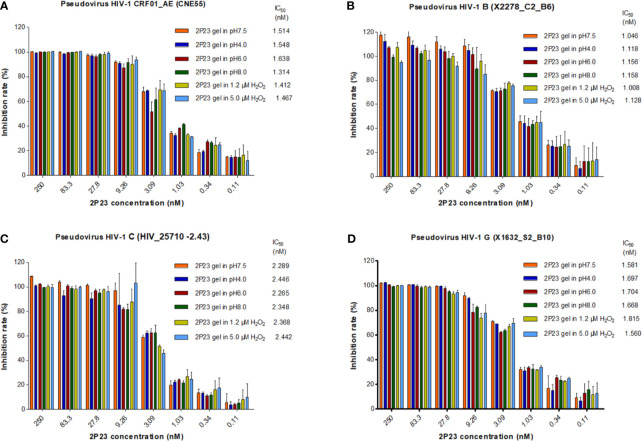
Antiviral activity of the 2P23 gel under different pH and H_2_O_2_ conditions. A 2P23 gel was prepared in complete growth medium (GM) at pH 7.5, 4.0, 6.0, and 8.0 or in the presence of 1.2 μM H_2_O_2_ and 5 μM H_2_O_2_ and incubated at 37°C for 48 h Single-round viral infection tests were performed using TZM-bl reporter cells and HIV-1 **(A)** pseudotyped HIV-1 CRF01_AE (CNE55), **(B)** pseudotyped HIV-1 B (X2278_C2_B6), **(C)** pseudotyped HIV-1 C (HIV_25710-2.43), and **(D)** pseudotyped HIV-1 G (X1632_S2_B10). The experiments were performed with an initial concentration of 2P23 peptide at 250 nM. These assays were performed in triplicate and repeated three times. Percent inhibition of the 2P23 gel in pH 7.5, pH 4.0, pH 6.0, pH 8.0, 1.2 µM H_2_O_2_ and 5 μM H_2_O_2_, and IC_50_ values were calculated as described in the text. Data are expressed as means ± standard deviations (SD).

The 2P23 gel is used in the vagina or rectum and therefore must be stable in the acidic pH of a healthy vagina (pH 3.5 to 4.5), near-neutral pH after ejaculation, or a healthy rectum (pH 7.4 to 8.4). The 2P23 gel should not be oxidized by H_2_O_2_ in the vaginal cavity. Under different pH and H_2_O_2_ conditions (**Figure 4**), the 2P23 gel always inhibited viral replication efficiently, indicating that pH and H_2_O_2_ had no significant effect on its antiviral activity. In conclusion, the high stability of the 2P23 gel under different pH and H_2_O_2_ conditions indicates that it is an ideal vaginal and rectal microbicide.

### 2P23 Formulated in Gel Is Stable at Different Temperatures

**Figure 5:**
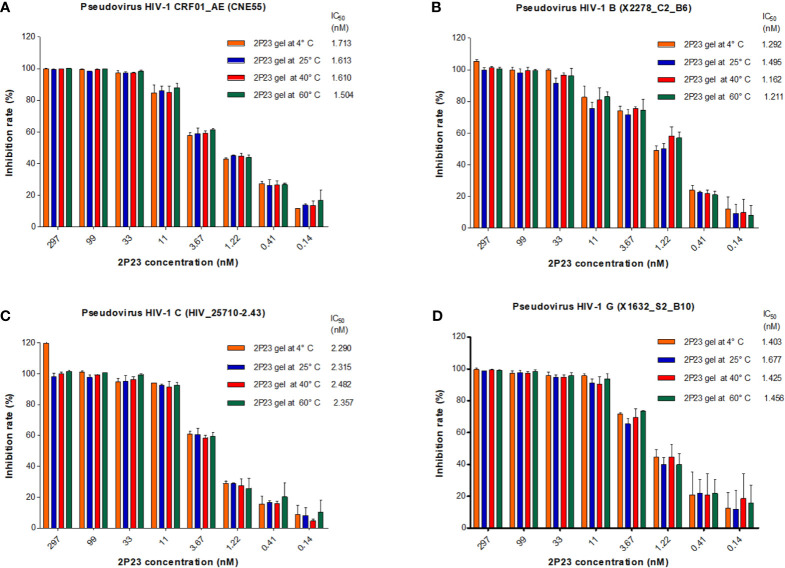
Antiviral activity and biological stability of the 2P23 gel at different temperature conditions. Stability of the 2P23 gel in complete growth medium was evaluated in a biological assay as a function of temperature. Thermal degradation studies were conducted at 60°C for 1 week, 40°C for 8 weeks, 25°C for 16 weeks, and 4°C for 24 weeks. Single-round viral infection tests were performed using TZM-bl reporter cells and HIV-1 **(A)** pseudotyped HIV-1 CRF01_AE (CNE55), **(B)** pseudotyped HIV-1 B (X2278_C2_B6), **(C)** pseudotyped HIV-1 C (HIV_25710-2.43), and **(D)** pseudotyped HIV-1 G (X1632_S2_B10). The experiments were performed with an initial concentration of 2P23 peptide at 297 nM. These assays were performed in triplicate and repeated three times. Percent inhibition of the 2P23 gel at 4°C, 25°C, 40°C, and 60°C and IC_50_ values were calculated as described in the text. Data are expressed as means ± standard deviations (SD).

**Figure 6:**
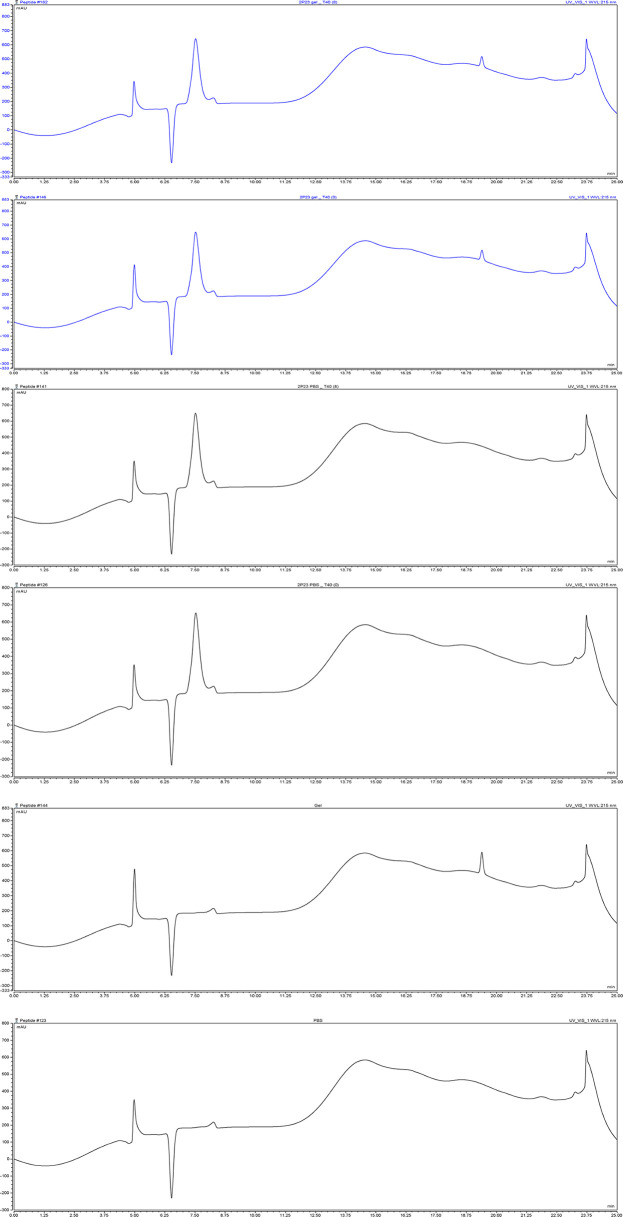
The content of 2P23 formulated in gel or in PBS was monitored by HPLC. Representative chromatograms of 2P23 formulated in gel or in PBS. The retention time (Ret. Time) was 7.5 min for 2P23. 2P23 gel_T40 (8), 2P23 gel_T40 (0): 2P23 in gel had been stored at 40°C for 8 weeks, 2P23 in gel at 40°C for 0 week; 2P23 PBS_T40 (8), 2P23 PBS_T40 (0): 2P23 in PBS had been stored at 40°C for 8 weeks, 2P23 PBS at 40°C for 0 week; Gel, PBS: Gel control, PBS control.

The 2P23 peptide must remain stable at different temperatures during the shelf life of the compound and in the human body during the manufacture of microbicides. To test their thermal stability, 2P23 gels were stored at 60°C for 1 week, 40°C for 8 weeks, 25°C for 16 weeks, and 4°C for 24 weeks. Antiviral activity was measured and compared to the control measured at 37°C for 48 h (**Figure 5**). The 2P23 gel showed the same antiviral activity when stored at 60°C for 1 week, 40°C for 8 weeks, 25°C for 16 weeks, and 4°C for 24 weeks. Together, these results highlight the very high thermal stability of the 2P23 gel. As shown in **Figure 6**, compared to the freshly prepared gel (0 weeks), 2P23 in the gel stored at 40°C for 8 weeks and 2P23 in PBS had the same peak shape and preservation time. At the end of the 8th week of storage at 40°C, the recoveries of 2P23 in gel and PBS were both 100%, indicating that there were no obvious drug loss or degradation products after a sufficient period of storage at relatively high temperature.

### The 2P23 Gel Does Not Significantly Irritate or Damage the Rectal or Vaginal Mucosa

**Figure 7:**
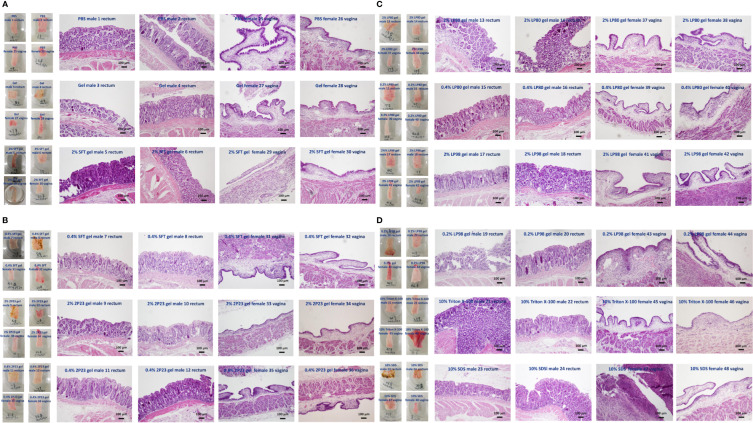
Histopathological analysis of vaginal or rectal epithelium in NZW rabbits of different groups after rectovaginal application of fusion inhibitor microbicides for 14 consecutive days. **(A)** PBS male rectum 1, 2; PBS female vagina 25, 26; gel male rectum 3, 4; gel female vagina 27, 28; 2% SFT gel male rectum 5, 6; 2% SFT gel female vagina 29, 30. **(B)** 0.4% SFT gel male rectum 7, 8; 0.4% SFT gel female vagina 31, 32; 2% 2P23 gel male rectum 9, 10; 2% 2P23 gel female vagina 33, 34; 0.4% 2P23 gel male rectum 11, 12; 0.4% 2P23 gel female vagina 35, 36. **(C)** 2% LP80 gel male rectum 13, 14; 2% LP80 gel female vagina 37, 38; 0.2% LP80 gel male rectum 15, 16; 0.2% LP80 gel female vagina 39, 40; 2% LP98 gel male rectum 17, 18; 2% LP98 gel female vagina 41, 42. **(D)** 0.2% LP98 gel male rectum 19, 20; 0.2% LP98 gel female vagina 43, 44; 10% Triton X-100 male rectum 21, 22; 10% Triton X-100 female vagina 45, 46; 10% SDS male rectum 23, 24; 10% SDS female vagina 47, 48.

The study was designed to have four animals per experimental group. Lifestyle observation involves assessing signs of irritation in the rectal and vaginal areas. After 14 days of rectal or vaginal administration of the 2P23 gel, no 2P23 gel-related changes with respect to physical signs or body weight were observed in animals. No significant abnormalities were found in the body surface, anus, or vagina by gross examination. Histopathological examination of the rectum and vagina is shown in **Figure 7**. No significant irritation was observed in the high or low doses of the 2P23 gel compared to the control group. At autopsy, rectal and vaginal irritation was assessed by the Eckstein method, which reflects the collective histopathological grading of four parameters within the vagina, including epithelial morphology, leucocyte infiltration, congestion, and edema. A mean irritation score of ≤8 was considered acceptable for clinical testing of rectal and vaginal products. The results of the rabbit model showed good safety profiles for the 2% 2P23 gel and 0.4% 2P23 gel. No gross anatomic pathology associated with 2P23 gel treatment was observed at any dose. There were few to no rectal or vaginal lesions on the 2P23 gel following once-daily dosing for 14 days. There was virtually no detectable rectal or vaginal irritation during the study. In the postmortem individual Eckstein score (maximum score 16) for the rectum and vagina of male and female rabbits at 4 mg/day, the 20 mg/day dose group was generally comparable between the two groups and ranged from none to minimal magnitude (ranging from 1 to 4 in the rectum and vagina). In rabbits treated with 100 mg/day SDS, epithelial disruption and vascular congestion were observed. Some tissues exhibited necrotic ulcerative granuloma. The inflammatory infiltrate had spread to the submucosa and may be severe. The histopathologic mean irritation scores are illustrated in **Table 4**. The total score obtained in these rabbit samples indicated the presence of average rectal and vaginal irritation. Taken together, these results suggest that 2P23 can be safely used as a microbicide at doses up to 20 mg/day (5,379,000 nM). After 14 days of continuous rectal and vaginal administration of the 2P23 gel, no 2P23 gel-related changes in physical signs or body weight were observed in rabbits. Histopathological examination found no irritation of the 2P23 gel to the rectal or vaginal mucosa, indicating that the product has good safety.

**Table 4:** Histopathological scores of combined rectal and vaginal irritation after 14 consecutive days of rectovaginal application of fusion inhibitor microbicides in NZW rabbits of different groups.

Group	PBS		Gel		2% SFT gel		0.4% SFT gel		2% 2P23-gel		0.4% 2P23-gel		2% LP80 gel		0.2% LP80 gel		2% LP98 gel		0.2% LP98 gel		10% Triton X-100			10% SDS	
Male ID	1	2	3	4	5	6	7	8	9	10	11	12	13	14	15	`16	17	18	19	20	21	22	23		24
Epithelial lesion	1	1	1	1	1	1	1	1	1	1	1	1	1	1	1	1	1	1	1	1	2	2	3		3
Inflammatory infiltrate	2	3	2	2	3	2	2	2	3	2	3	3	2	2	2	2	2	3	3	3	2	3	2		3
Vascular congestion	0	0	0	0	0	0	0	0	0	0	0	0	0	0	0	0	0	0	0	0	1	1	2		2
Edema/fibrosis	0	0	0	0	0	0	0	0	0	0	0	0	0	0	0	0	0	0	0	0	1	1	2		2
Total Score ^*^	3	4	3	3	4	3	3	3	4	3	4	4	3	3	3	3	3	4	4	4	6	7	9		10
Female ID	25	26	27	28	29	30	31	32	33	34	35	36	37	38	39	40	41	42	43	44	45	46	47		48
Epithelial lesion	1	1	1	1	1	1	1	1	1	1	1	1	1	1	1	1	1	1	1	1	2	2	4		3
Inflammatory infiltrate	2	3	3	3	2	2	2	2	2	2	2	2	2	2	2	2	3	2	3	2	2	2	4		2
Vascular congestion	0	0	0	0	0	0	0	0	0	0	0	0	0	0	0	0	0	0	0	0	1	1	4		2
Edema/fibrosis	0	0	0	0	0	0	0	0	0	0	0	0	0	0	0	0	0	0	0	0	1	1	4		2
Total Score ^*^	3	4	4	4	3	3	3	3	3	3	3	3	3	3	3	3	4	3	4	3	6	6	16		9

*The score for each lesion was as follows: 0 (no change), when there was no injury or the observed change was within the normal range; 1 (minimum), when the changes are sparse but exceed those considered normal; 2 (mild), when injuries were identifiable but with no severity; 3 (moderate), severe injury can increase the severity; 4 (very severe), very severe injuries occupied most of the analyzed tissue. These scores were added up to determine levels of vaginal and rectal irritation as minimum (1–4), mild (5–8), moderate (9–11), and severe (12–16).

### The 2P23 Gel Does Not Significantly Trigger Rectal or Vaginal Mucosa Secretion of Inflammatory Cytokines

To further evaluate the inflammatory potential of the 2P23 gel on the rectal and vaginal mucosa, RL and CVL were collected from each test animal after 14 consecutive days of treatment, and the presence of 14 cytokines was measured and quantified by ELISA. We measured the secretion of inflammatory cytokines triggered by these candidate microbicides at local sites of the mucosa. Compared to PBS, the 2P23 gel did not induce obvious enhancement of cytokine production (**Table 5**). Taken together, the HIV membrane fusion inhibitor 2P23 gel has the potential to be developed as a safe and effective anti-HIV microbicide for the prevention of sexual transmission of HIV.

**Table 5:** Inflammatory cytokines in the rectum and vagina after 14 consecutive days of rectovaginal application of fusion inhibitor microbicides in male and female NZW rabbits.

Group	PBS	Gel	2% SFT gel	0.4% SFT gel	2% 2P23-gel	0.4% 2P23-gel	2% LP80 gel	0.2% LP80 gel	2% LP98 gel	0.2% LP98 gel	10% Triton X-100	10% SDS
**Male**												
IL-4 (pg/ml)	8.495	6.260	6.445	22.932	6.819	5.235	12.283	6.290	12.438	8.805	1.354	4.707
IL-5 (pg/ml)	4.926	3.618	3.727	13.374	3.945	3.019	7.142	3.636	7.233	5.108	0.748	2.710
IL-6 (pg/ml)	13.547	41.251	22.197	5.025	34.114	22.852	29.582	17.326	34.724	141.775	8.398	20.300
IL-8 (pg/ml)	16.489	15.207	16.864	40.329	15.426	18.366	28.503	14.674	28.909	11.858	9.075	11.953
IL-10 (pg/ml)	46.516	22.575	54.875	28.816	32.740	30.533	64.171	32.449	67.114	27.322	23.243	28.215
IL-17 (pg/ml)	27.447	28.783	74.516	52.028	42.427	54.772	64.625	41.380	56.865	19.253	51.379	34.919
IFN-γ (pg/ml)	92.078	29.989	208.731	55.217	36.477	56.667	138.394	213.390	168.524	74.476	69.886	33.475
MCP1 (pg/ml)	316.515	290.692	458.542	518.796	341.477	312.211	359.553	298.438	392.262	265.729	291.552	302.742
IL1a (pg/ml)	18.121	16.398	16.178	21.786	17.181	14.425	22.976	17.964	18.497	14.894	9.538	13.234
IL1RA (pg/ml)	73.422	29.354	47.695	16.805	47.783	25.346	49.200	52.052	46.470	47.170	22.021	42.672
E-SELE (pg/ml)	420.948	844.272	1,689.902	719.662	647.309	1,502.838	1,475.119	700.284	1,029.177	1,565.472	361.133	810.675
ICAM1 (pg/ml)	5.415	8.572	7.295	3.576	9.659	7.947	7.082	19.329	7.302	18.154	8.012	7.298
VEGFA (pg/ml)	82.420	10.661	70.077	14.887	7.144	10.931	45.728	54.893	34.941	14.955	20.231	7.516
PDGFA (pg/ml)	29.784	18.279	67.158	18.247	12.752	14.423	59.220	54.271	82.101	22.135	24.482	10.277
**Female**												
IL-4 (pg/ml)	5.297	5.856	13.059	7.905	7.098	4.024	14.953	6.290	4.148	3.837	5.577	13.463
IL-5 (pg/ml)	3.055	3.382	7.597	4.581	4.109	2.310	8.705	3.636	2.383	2.201	3.219	7.833
IL-6 (pg/ml)	13.510	12.290	29.680	8.894	5.229	2.677	7.780	9.308	10.031	3.278	26.947	5.869
IL-8 (pg/ml)	29.035	27.346	33.196	34.134	18.304	20.650	26.375	16.051	22.840	69.174	22.496	108.345
IL-10 (pg/ml)	61.898	63.124	54.006	49.592	76.945	70.815	34.879	53.761	60.760	79.017	20.613	121.529
IL-17 (pg/ml)	51.703	53.761	48.274	64.950	87.222	101.299	73.288	60.871	82.312	54.843	80.039	150.895
IFN-γ (pg/ml)	267.266	210.009	179.637	282.037	210.147	160.310	182.122	163.071	194.892	339.087	199.793	450.771
MCP1 (pg/ml)	166.741	402.592	327.705	425.832	188.26	253.678	398.288	177.931	130.588	407.756	486.947	289.831
IL1a (pg/ml)	13.986	14.801	15.928	23.133	14.268	12.764	23.853	16.366	10.446	14.643	10.227	26.955
IL1RA (pg/ml)	52.771	50.180	121.304	37.387	51.335	53.611	56.183	52.718	82.872	77.166	51.388	156.026
E-SELE (pg/ml)	1,694.582	1,813.372	1,258.297	1,631.046	1,879.006	2,232.737	1,933.241	1,750.316	1,510.216	2,038.053	1,991.377	1,421.484
ICAM1 (pg/ml)	9.161	13.541	9.722	41.344	9.672	7.898	10.505	7.948	6.441	9.798	35.134	10.787
VEGFA (pg/ml)	62.401	81.947	46.100	65.105	61.116	45.560	62.062	38.762	41.806	88.541	58.647	88.609
PDGFA (pg/ml)	125.903	85.315	58.031	64.619	65.776	65.198	69.954	34.315	58.352	159.292	194.449	99.037

### The 2P23 Gel Is Not Associated With Changes to the Rectal or Vaginal Microbiota

**Figure 8:**
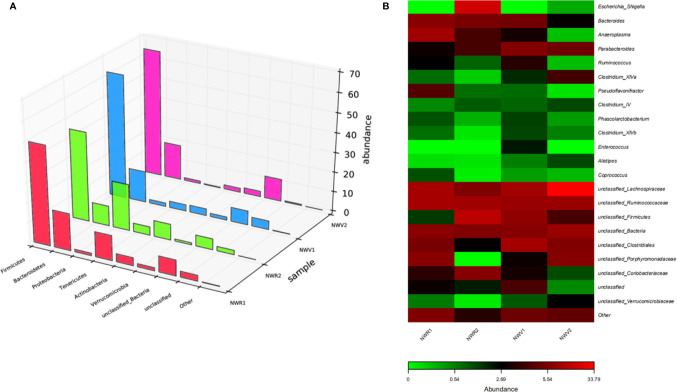
Diversity of microbial community composition across the rectum and vagina. **(A)** The composition of the microbiota was visualized by 3D bar plots. The horizontal axis represents the bacterial colonies. The vertical axis represents the relative abundance ratio. The *z*-axis represents the sample. **(B)** Heat map showing the top 23 similarly abundant genera in the rectum versus vagina. Each column represents data from a single animal.

Maintenance of the mucosal barrier is critical for preventing microbial invasion, including HIV, and bacterial diversity in the rectum and vagina is closely associated with mucosal inflammation, which negatively affects local vulnerability to HIV infection. Microbial communities in the rectum and vaginal mucosal sites may influence the efficacy of topical HIV drugs (50, 51). We evaluated changes in the composition of the microbiota in vaginal and rectal samples before and after 2P23 gel administration using bacterial 16S rRNA sequencing. We collected pairs of RL and CVL samples from 48 NZW rabbits and simultaneously treated the extracted DNA and sequenced 16S ribosomal RNA genes, providing a strong opportunity for comparative evaluation of these different mucosal compartments. In addition, the mean sequencing depth of the RL and CVL samples was the same, and the number of high-quality reads was similar, suggesting that the irrigation sampling technique effectively restored and amplified bacterial DNA. We obtained 937 million high-quality reads from RL samples and 958 million bacterial reads from CVL samples. Silva 132 was used as a reference database to classify high-quality reads (52). Before investigating whether the immune response after 2P23 gel rectovaginal application correlates with changes in the mucosal microbiome profile, we evaluated the bacterial composition similarities and differences in the rectal and vaginal compartments at baseline (Day 0 of 2P23 gel rectovaginal application). **Figure 8** shows the composition of the microbiota clustered by similarity in the relative abundance of organisms. At the phylum level, the bacterial communities in the rectum and vagina were composed of *Firmicutes*, *Bacteroidetes*, *Tenericutes*, *Actinobacteria*, *Verrucomicrobia*, and *Proteobacteria* (**Figure 8A**). An assessment of microbial composition at the genus level revealed that the rectal and vaginal microbiota are composed of similar phylotypes (**Figure 8B**).

**Figure 9:**
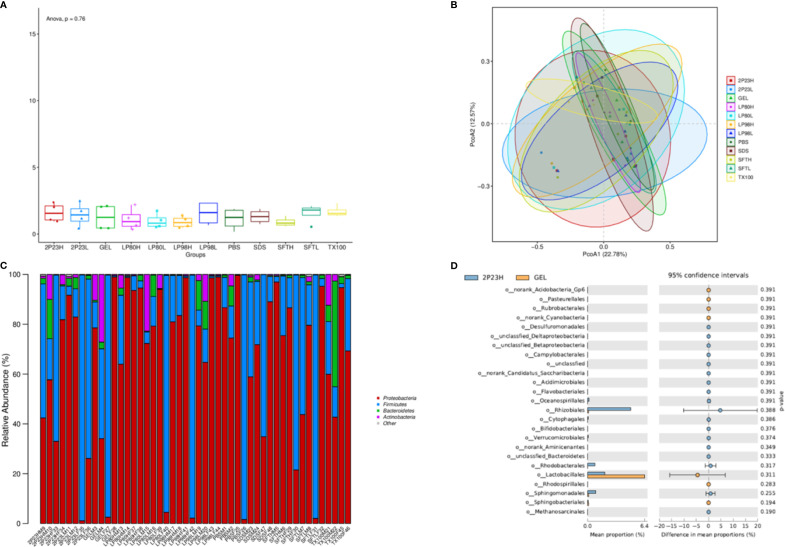
Microbial changes following 2P23 gel administration. **(A)** Rectal and vaginal microbiota comparison following 2P23 gel administration was analyzed using shannon_alpha_diversity_test. **(B)** 16S rRNA sequence data from paired rectal and vaginal samples showed unique clustering evident from a principal coordinate analysis (PCoA) plot based on unweighted UniFrac distances between bacterial communities across each mucosal site at day 14. **(C)** Histogram of the relative abundance of taxonomic classification at the phylum level. The horizontal axis is the serial number of each sample, and the vertical axis is the relative abundance ratio of species. Colors correspond to species names at this taxonomic level, and different color block widths represent the relative abundance ratio of different species. **(D)** Diagram of difference analytic results at the genus level list only the 25 with the lowest *p*-values. The figure on the left shows the abundance ratio of different species in the two groups of samples; the middle shows the 95% confidence interval, the proportion of differences in species abundances; the rightmost value is a *p*-value, where a *p*-value < 0.05 indicates a significant difference.

We next evaluated changes in the composition of the microbiota in rectal and vaginal samples in response to 2P23 gel rectovaginal application by bacterial 16S rRNA sequencing. Due to the rectovaginal application of the 2P23 gel, no significant change in Shannon alpha diversity of the rectal or vaginal microbiota was observed (**Figure 9A**). Comparison of microbial communities at the phylum level showed no significant differences between 2P23-gel rectovaginal-applied samples (**Figure 9B**). Weighted PCoA analysis showed slight differences among animals in most sample clusters and rectal and vaginal microbiomes (**Figure 9C**). We detected noticeable variation in the relative abundance of *Rhizobiales*, which belongs to *Proteobacteria*, and *Lactobacillales*, which belongs to *Firmicutes*, in the 2P23 gel high-dose and gel groups (**Figure 9D**). Overall, the data demonstrated that the rectal and vaginal microbiome is highly polymicrobial and remains largely stable during the course of HIV membrane fusion inhibitor 2P23 microbicide rectovaginal application. Rabbits are relevant models for studying pathogenesis and verifying strategies to prevent the spread of infection. Our study provides a detailed characterization of the rectal and vaginal microbiota of male and female NZW rabbits and opens up new prospects for establishing this animal model. Overall, our data suggest that the safety and ability of 2P23 gels to inhibit HIV infection is independent of rectal and vaginal microbiome differences.

## Discussion

The main findings of this study include the following: (1) the inhibitory effect of 2P23 gel on HIV infection was stable and effective against a broad spectrum of HIV strains, as evidenced by IC_50_ values ranging from 0.5908 to 2.084 nM *in vitro*; (2) the potency of anti-HIV infection of 2P23 gel was stable when kept at different temperatures, different pH values, and different H_2_O_2_ concentrations; (3) 2P23 gel did not induce cell death in PBMCs or immune cells *in vitro*; and (4) when administered to rabbits, 2P23 gel did not influence physical rectal or vaginal bacterial expansion. Moreover, it induced neither morphological changes nor inflammatory cytokine production in the rectal or vaginal mucosa.

Due to the deleterious destruction of the immune defense system by HIV infection, patients experience a lifelong threat for immunodeficiency-related diseases. If better HIV prevention is attained, a lower incidence of HIV infection is achieved. To date, there are three main strategies for HIV prevention: lifestyle modification, condom use, and microbicides. Given the sustained high rate of HIV infection, a gap remains for education, improved compliance, and recognition of condom usage.

Microbicides circumvent viral infection of targeted cells and therefore hold promise for anti-HIV infection. However, the therapeutic effect of topical PrEP application is dependent on the ARV regimen. At present, the most promising microbicides are nucleotide reverse transcriptase inhibitors, such as tenofovir. By inhibiting RNA transcription, tenofovir prohibits viral replication, such as hepatitis and HIV infection, in host cells. In a randomized, placebo-controlled trial, 12,320 women with no HIV infection were assigned to oral administration of tenofovir disoproxil fumarate (TDF), tenofovir-emtricitabine (TDF-FTC), or 1% tenofovir (TFV) vaginal gel and followed up for 12–36 months. By intention-to-treat analysis, the incidence of HIV infection was comparable among all groups (53, 54). Likewise, resistance to tenofovir is commonly observed in low- and middle-income countries (55, 56). These data suggest that new drugs are needed for HIV prevention.

Previously, we reported a synthetic HIV membrane fusion inhibitor peptide called 2P23 that has strong activity against HIV-1, HIV-2, and SIV (23). In this study, we further evaluated its physical and biological as well as its toxic effects *in vitro* and *in vivo*. When formulated into a gel, 2P23 was stably released, maintained, and blocked HIV infection *in vitro*, independent of temperature (25°C–60°C), pH (4.0–8.0), and H_2_O_2_ concentration. We developed a 2P23 gel dosage form and examined its physical and chemical properties to meet the requirements of rectal and vaginal administration environments and reduce irritation (57–60). HEC was added to the preparation as a gel matrix so that the preparation had a suitable viscosity and could be retained in the rectum and vagina. After 14 days of continuous rectovaginal application of the 2P23 gel, no 2P23 gel-related changes in physical signs or body weight were observed in rabbits. Histopathological examination found no irritation of the 2P23 gel to the rectal or vaginal mucosa, indicating that the product has good safety.

Notably, 2P23 had no effect on physical bacterial expansion in the rectum or vagina *in vitro* or *in vivo*, which is crucial for biological defense and function in the body. This is important because rectovaginal application of microbicides should protect the normal rectal and vaginal microbiota, particularly *Lactobacillus*, *Bifidobacterium*, *Enterobacter*, and *Enterococcus*, which are essential for maintaining a balance of a healthy rectal and vaginal bacterial ecosystem. Loss of rectal and vaginal mucosal integrity and abnormal rectal and vaginal microbiota, e.g., the abundance of γ-*Proteobacteria*, the absence of *Lachnospiraceae* and *Ruminococcaceae*, and the reduction of alpha diversity are thought to be mechanisms that lead to chronic inflammation and increased morbidity and mortality during antiretroviral therapy for HIV disease (61–63). The vaginal microflora dominated by *Lactobacillus* may be enhanced, and inflammatory cytokines may attenuate the protective effect of vaginal TFV gel in healthy HIV-negative women (64–66).

This is the first study to evaluate the rectal and vaginal use of the 2P23 gel. The safety and stability of the 2P23 gel indicate that the HIV membrane fusion inhibitor 2P23 peptide is an optimal microbicide for HIV prevention.

## Conclusions

In conclusion, this multiplatform study, which combines cytokine and microbiome monitoring techniques, provides comprehensive information on the rectal and vaginal mucosal environment at a systematic level, providing an additional tool for preclinical microbicide safety evaluation. In general, the 2P23 microbicide gel did not have any significant effect on mucosal cytokines and had only a small effect on the microbiome, indicating its tolerability and suitability as a candidate microbicide. This study highlights the use of 2P23 gel as a suitable candidate for PrEP, which may offer the potential for better adoption and effective HIV prevention by men and women in end users. The 2P23 gel did not cause cellular or bacterial toxicity, nor did it alter the microbiome of the rectum or vagina. The versatility of gel formulations offers the potential to create products that protect men and women from HIV infection and enhance mucosal health.

## Data Availability Statement

The datasets presented in this study can be found in online repositories. The names of the repository/repositories and accession number(s) can be found below: NCBI SRA; PRJNA726948.

## Ethics Statement

All animal experiments were performed according to the guidelines and protocols approved by the Ethics Committee for Animal Experimentation of National Institutes for Food and Drug Control (NIFDC; Beijing, China). All animal experiments were reviewed and approved by the Institutional Animal Care and Use Committee (IACUC) of NIFDC (approved on 2 August 2019; protocol No. 2019 (B) 010). To ensure personnel safety and animal welfare, the study of animals were conducted in strict accordance with the recommendations for conducting experiments were in accord with the highest scientific, humane, and ethical principles as stated in the Guide for the Care and Use of Laboratory Animals. Written informed consent was obtained from the owners for the participation of their animals in this study.

## Author Contributions

ZG and YH conceived the study and designed the experiments. ZG, RF, XL, and JW performed the experiments. ZG and YH wrote the article. All authors contributed to the article and approved the submitted version.

## Funding

This study was supported by the grant from the CAMS Innovation Fund for Medical Sciences (2017-I2M-1014). The funders had no role in study design, data collection and analysis, decision to publish, or preparation of the manuscript.

## Conflict of Interest

The authors declare that the research was conducted in the absence of any commercial or financial relationships that could be construed as a potential conflict of interest.

## Publisher’s Note

All claims expressed in this article are solely those of the authors and do not necessarily represent those of their affiliated organizations, or those of the publisher, the editors and the reviewers. Any product that may be evaluated in this article, or claim that may be made by its manufacturer, is not guaranteed or endorsed by the publisher.
